# Correction to “Gap Between Patients With Schizophrenia and Their Psychiatrists on the Needs to Psychopharmacological Treatment: A Cross‐Sectional Study”

**DOI:** 10.1002/npr2.70024

**Published:** 2025-05-12

K. Takahashi, R. Yamazawa, T. Suzuki, M. Mimura, and H. Uchida, “Gap between patients with schizophrenia and their psychiatrists on the needs to psychopharmacological treatment: A cross‐sectional study,” *Neuropsychopharmacology Reports* 40, no. 3 (2020): 232–238, https://doi.org/10.1002/npr2.12118.

In the last row of Table 1, the value listed for “Morisky Simplified Self‐Report Measure of Adherence” is “1.6 ± 1.2.” This is incorrect. It should have been: “2.4 ± 1.2.”

In the “Methods” section, the third sentence in the subsection “Statistical analyses” is as follows:

“Preferences of dosing frequency, timing, and dosage form were compared between patients who showed good medication adherence (i.e., 0‐2 in the Morisky Simplified Self‐Report Measure of Adherence) and those who did not (i.e., 3 and 4 in the Morisky Simplified Self‐Report Measure of Adherence) by a chi‐squared test.”

The correct sentence should be:

“Preferences of dosing frequency, timing, and dosage form were compared between patients who showed good medication adherence (i.e., 3 and 4 in the Morisky Simplified Self‐Report Measure of Adherence) and those who did not (i.e., 0‐2 in the Morisky Simplified Self‐Report Measure of Adherence) by a chi‐squared test.”

We apologize for this error.

